# RNA-seq and microarray complement each other in transcriptome profiling

**DOI:** 10.1186/1471-2164-13-629

**Published:** 2012-11-15

**Authors:** Sunitha Kogenaru, Qing Yan, Yinping Guo, Nian Wang

**Affiliations:** 1 Citrus Research and Education Center, Department of Microbiology and Cell Science, Institute of Food and Agricultural Sciences, University of Florida, 700 Experiment Station Road, Lake Alfred, 33850, USA

**Keywords:** RNA-seq, Microarray, Transcriptome profiling, Pathogenic bacteria, Virulence, Type 3 secretion system, Effectors, HrpX, Xanthomonas, Citrus canker disease

## Abstract

**Background:**

RNA-seq and microarray are the two popular methods employed for genome-wide transcriptome profiling. Current comparison studies have shown that transcriptome quantified by these two methods correlated well. However, none of them have addressed if they complement each other, considering the strengths and the limitations inherent with them. The pivotal requirement to address this question is the knowledge of a well known data set. In this regard, HrpX regulome from pathogenic bacteria serves as an ideal choice as the target genes of HrpX transcription factor are well studied due to their central role in pathogenicity.

**Results:**

We compared the performance of RNA-seq and microarray in their ability to detect known HrpX target genes by profiling the transcriptome from the wild-type and the *hrpX* mutant strains of γ-Proteobacterium *Xanthomonas citri* subsp. *citri*. Our comparative analysis indicated that gene expression levels quantified by RNA-seq and microarray well-correlated both at absolute as well as relative levels (Spearman correlation-coefficient, r_s_ > 0.76). Further, the expression levels quantified by RNA-seq and microarray for the significantly differentially expressed genes (DEGs) also well-correlated with qRT-PCR based quantification (r_s_ = 0.58 to 0.94). Finally, in addition to the 55 newly identified DEGs, 72% of the already known HrpX target genes were detected by both RNA-seq and microarray, while, the remaining 28% could only be detected by either one of the methods.

**Conclusions:**

This study has significantly advanced our understanding of the regulome of the critical transcriptional factor HrpX. RNA-seq and microarray together provide a more comprehensive picture of HrpX regulome by uniquely identifying new DEGs. Our study demonstrated that RNA-seq and microarray complement each other in transcriptome profiling.

## Background

Transcriptome of an organism represents the entire repertoire of transcripts encoded by the genes as a phenotypic response to the condition in which they exist. The sheer ability to simultaneously quantify the expression levels for a vast number of genes has revolutionized the biomedical research, facilitating the analysis of global gene expression patterns at the genome-wide scale [1]. In the past decade, there has been a tremendous progress in the development of methods to deduce and quantify the gene expression levels at the whole transcriptome level [1]. Among the several transcriptome profiling methods, RNA-seq and DNA microarray stand out as the two widely used genome-wide gene expression quantification methods [1-17].

RNA-seq method involves the conversion of isolated transcripts into the complementary DNA (cDNA), which is then directly sequenced in a massively parallel deep-sequencing-based approach [18]. By mapping the resulting short sequencing reads onto the reference genome, the expression levels of genes relative to the condition of interest or absolute levels can be quantified [9,11]. This method has been implemented in different platforms like Illumina’s Genome Analyzer, Roche 454 Genome Sequence, and Applied Biosystems’ SOLiD [4]. On the other hand, microarray is based on the hybridization of specimen target strands onto the immobilized complementary probe strands. For example, in a two-color microarray, transcripts extracted from different conditions are labeled with distinct fluorescent dyes while being converted to cDNA. These labeled samples are then hybridized to the immobilized complementary probe strands in an array representing the genes. By measuring the light intensity of the distinct fluorescent dyes, the relative abundance of each transcript in the two different conditions can be measured [8,12,13,17,19,20]. Affymetrix and Agilent are the two prevalent platforms in microarray technology [2,14].

Even though, initially microarray has been instrumental in whole transcriptome analysis, currently RNA-seq is becoming a preferred method of choice, since it is considered to effectively surmount the limitations of microarray [1,21-23]. RNA-seq technology, unlike microarray, does not depend on the prerequisite knowledge of the reference transcriptome [24]. Further, RNA-seq data contains very low background signal, a higher dynamic range of expression levels, and also relatively small amount of total RNA required for quantification, when compared to microarray [1,23]. Despite these advantages, the efficiency of RNA-seq is marred with the problem of overwhelming amount of ribosomal RNA (rRNA) in the data, short reads, less base accuracy, and variation of read density along the length of the transcript, posing a challenge for this high-throughput method [21,25,26]. However, in spite of their strengths and limitations, RNA-seq and microarray have become the default popular methods of choices for genome-wide transcriptome studies [1,2,23].

Currently several studies have been conducted to compare the performance of RNA-seq and microarray in quantifying the expression level of genes, by focusing on various aspects like reproducibility, accuracy, statistical issues, technical and biological variabilities [1,15,21,27-30]. The main conclusion from these studies has been that the expression levels quantified by these two methods correlated to a large extent, and overall favored the RNA-seq because of high reproducibility, accuracy, and dynamic range [27,29]. However, none of these comparison studies have addressed if these two methods complement each other in transcriptome profiling given the strengths and limitations associated with them. In order to address this question, we require an already well characterized dataset. The HrpX regulome from *Xanthomonas citri* subsp*. citri* (Xcc) serves as an ideal data model in this regard [31-33]. Xcc is a causal agent of citrus canker, one of the serious and destructive diseases in citrus that is resulting in significant losses to citrus industry worldwide [34], while HrpX is a key global transcription factor that regulates the expression of *hrp* (hypersensitive response and pathogenicity) cluster of genes, which are considered as the major pathogenicity factors [31,35]. HrpX contains AraC-type of DNA binding domain, which specifically recognizes the plant-inducible promoter (PIP) box (TTCGC-N15-TTCGC) and imperfect PIP box (TTCGC-N8-TTCGT) present in the cis-regulatory regions of *hrp* gene cluster [36-38]. Since HrpX has a key role in pathogenicity, tremendous progress has been made in cataloguing the target genes of HrpX [39-45]. We therefore assessed the performance of RNA-seq and microarray in their ability to detect known HrpX target genes. We chose Illumina and Agilent as the corresponding platforms for RNA-seq and microarray, as they are the most popular platforms for these technologies [2,4].

## Results

In order to uncover the regulome of HrpX transcription regulator by profiling the wild-type and the *hrpX* mutant strains transcriptome, we had designed a microarray chip covering the whole genome under Agilent platform in our previous study [33]. Here, we conducted genome-wide transcriptome profiling of these two strains by RNA-seq and compared the results to the previously published microarray data, to assess the performance of these two methods. Further, to avoid technical variation associated with RNA isolation, we used the aliquots from the same total RNA samples used for microarray experiments also for RNA-seq.

We obtained 16,431,283, 17,289,220, 18,124,120 sequence reads for the wild-type and 15,084,955, 17,831,920, and 18,115,115 for the *hrpX* mutant strain with a median sequence length of 74-base pairs (bp) (Additional file 1: Table S1). Raw reads often have high sequencing errors, especially in the 3^′^ end where there is a high chance of sequencing errors to occur [46]. We therefore filtered the reads for high quality ones by trimming off the base pairs with low quality score assigned to them during down-line processing of RNA-seq. More than 90% of the reads passed the quality filter, as a result, the median sequence length of quality filtered reads subsequently dropped to 68-bp (Additional file 1: Table S1). We then mapped these high quality trimmed reads on to the Xcc genome. Approximately more than 90% of the reads could be mapped on to the reference genome, indicating good sequence coverage (Additional file 1: Table S1). Overall ~97% of the annotated genes had more than one read mapped, while merely ~3% of the annotated genes had no reads mapped, indicating good sequencing depth. Further, we also observed a difference in the sequence coverage between the chromosome and the two endogenous plasmids of Xcc. Annotated coding genes from the chromosome with a size of 5.18 mega base pairs (Mb) had 98% sequence coverage, whereas, it was 78% for plasmid pXAC64 with a size of 0.06 Mb, and relatively lower with only 62% sequence coverage for plasmid pXAC33 with a size of 0.03 Mb (Additional file 1: Table S2).

### Comparison at absolute levels of expression

**Figure 1:**
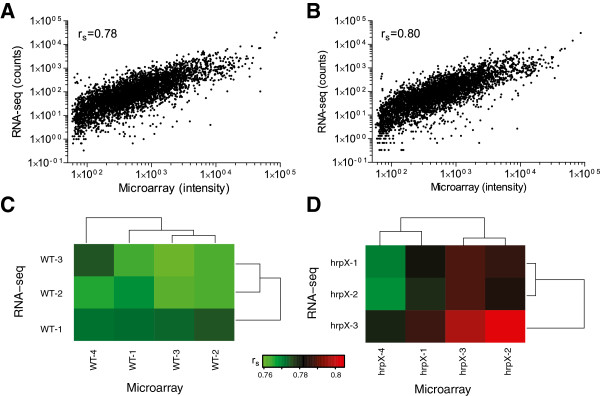
**Comparison of absolute levels of gene expression by RNA-seq and microarray. **Upper panel shows for the (**A**) wild-type and (**B**) *hrpX* mutant, the correlation between normalized fluorescence intensities of Agilent microarray with the RNA-seq counts from Illumina. Each dot represents the average values for each gene from all the biological replicates. Spearman’s correlation coefficient (r_s_) is indicated for each comparison. Lower panel shows r_s_ between normalized fluorescence intensities of Agilent microarray with the RNA-seq counts from Illumina for all the combination of biological replicates for the (**C**) wild-type, and (**D**) the *hrpX* mutant. The r_s_ values are plotted in the form of a heat map, where green color represents low r_s_ value, while red represents highest r_s_ value. The dendrogram provides a hierarchical clustering.

RNA-seq had coverage for 4323 genes with one or more reads mapped, while by microarray 4349 genes were assigned the fluorescence intensity values after the background correction. Among these 4312 genes (~99% of the total genes) were common to both methods, while merely 37 (0.8%) and 11 genes (0.2%) were uniquely called by microarray and RNA-seq respectively (Additional file 1: Tables S2 and S3; Additional file 2: Figure FS1). We compared the absolute levels of gene expression in terms of RNA-seq counts and microarray fluorescence intensities for all the listed genes called by both the methods. These two independent measures of transcript abundance associated with each gene for all the biological replicates from the wild-type and the *hrpX* mutant strains were compared separately. The resulting correlation was mapped as a scatter plot, with an average number of counts from Illumina sequencing against the normalized fluorescence intensities from Agilent arrays for each gene in the wild-type (Figure 1A) as well as in the *hrpX* mutant (Figure 1B). Absolute levels of gene expression correlated well, when estimated in terms of Spearman’s correlation coefficient (r_s_) with 0.78 (p-value < 0.0001) for the wild-type and 0.80 (p-value < 0.0001) for the *hrpX* mutant strain. This is in agreement with the previous reports that expression levels measured by microarray and RNA-seq had correlations ranging between 0.62 and 0.8 for prokaryotic and eukaryotic datasets [18,28,29]. However, there seems to be little or no correlation for the genes with low level of expression. We further estimated the correlation for the subset of genes with fluorescence intensity values ≤100 assigned by microarray (~360 genes) with the corresponding expression levels determined by RNA-seq. This subset of genes revealed a very poor r_s_ of 0.2 (p-value <0.0002) and 0.3 (p-value <0.0001) for the wild-type and the *hrpX* mutant strains respectively. Although the expression levels of these genes did not change much according to microarray, RNA-seq reported them to have different expression levels. This may be attributed to the high sensitivity of RNA-seq method.

We further estimated the correlation between all the combinations of biological replicates for the wild-type and the *hrpX* mutant strains independently. The resulting r_s_ values of these comparisons are represented in the form of heat maps, for the wild-type (Figure 1C) and the *hrpX* mutant strains (Figure 1D), which provide a global view of these correlations. Overall, on an average the wild-type with r_s_ = 0.76 (p-value < 0.0001) and the *hrpX* mutant with r_s_ = 0.78 (p-value < 0.0001) were observed for the biological replicates from all the correlation combinations. This level of comparison strongly suggested that not only the absolute level of gene expressions determined by RNA-seq and microarray highly correlated, but were also highly reproducible, in spite of the technical as well as the biological variability associated with the quantifications.

### Comparison at relative levels of expression

**Figure 2:**
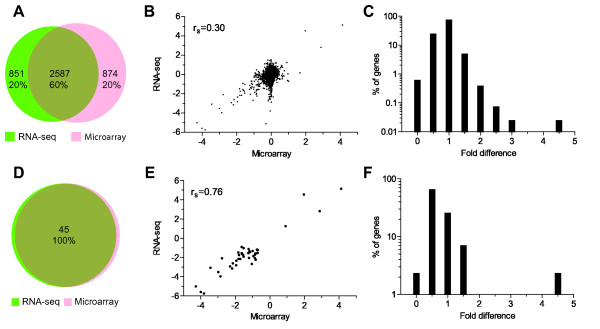
**Qualitative and quantitative comparison of relative levels of gene expression in the *hrpX *mutant with respect to the wild-type strain, determined by RNA-seq and microarray methods. **(**A**) Venn diagram showing the qualitative agreement in the log_2_ fold-change values for expression of 4312 genes by RNA-seq and microarray. (**B**) Scatter plot showing the relative expression levels of genes in terms of log_2_FCs, determined by RNA-seq and microarray. Correlation between the two methods is shown by Spearman’s correlation coefficient (r_s_). (**C**) Frequency histogram showing the percentage of genes with the fold difference between RNA-seq and microarray, with a bin width of 0.5. The lower panel **D**, **E**, and **F** are same as **A**, **B**, and **C** respectively, but for only those genes that have passed the statistical cut-off threshold (FDR ≤ 5% and absolute log_2_ fold-change ≥ 0.6).

We also compared the performance of these two methods at relative level of gene expression. For this purpose, we first computed the relative expression level of genes in terms of fold-change (FC) for the *hrpX* mutant in relation to the wild-type strain, along with p-values to denote the statistical significance and false discovery rate (FDR), for having a good control over the false positives rate. We compared the relative expression levels for 4312 consensus genes both qualitatively and quantitatively, after transforming the FC values to logarithm base 2 (log_2_) scale without any statistical cut-off thresholds (Additional file 1: Table S2). For the 2587 (~60% of the consensus) genes, the expression levels agreed qualitatively, while 1725 (~40%) genes disagreed between the two methods (Figure 2A). At this point, our comparison was exclusively focused on whether the gene of interest is up- or down-regulated based on the sign of the log_2_ transformed FC values, but not necessarily on the FC magnitude. We further illustrated the quantitative relationship of log_2_FC between RNA-seq and microarray in the form of a scatter plot as shown in Figure 2B. Genes with no change in expression levels in the wild-type and the *hrpX* mutant strains (FC = 1) clustered around log_2_FC of zero (log_2_ of one is zero) in the scatter plot (Figure 2B). The r_s_ between the log_2_FCs determined by RNA-seq and microarray was found to be 0.30 (p-value < 0.0001) (Figure 2B). This lower correlation value indicated that the magnitude of FCs between the two methods differed largely that might be due to the background noise resulting from the many imperfections, which are inherent to the high-throughput technologies [47,48].

The correlation coefficient provides an overall estimate of correlation between the expression levels determined by RNA-seq and microarray methods. However, this does not zoom into the data in a detailed manner. For instance, no information is provided about how much of FC magnitude that actually differs between the two methods for a given gene. In order to get an insight into this aspect, we computed the fraction of genes deviating in their FC magnitude values by dividing the FC magnitude value determined by RNA-seq with that of microarray (Figure 2C). Here, the fold difference of one represents the fraction of genes that are determined to have a FC magnitude of ± 0.5 (bin width) by both RNA-seq and microarray methods. When we plotted this frequency as a histogram for the whole 4312 consensus genes, more than 75% of genes were found to have FC magnitude values ± 0.5 by RNA-seq and microarray methods. Since it is a relative expression comparison, genes whose expression values did not change much in the wild-type and the *hrpX* mutant strains, tend to have FC values = 1. Subsequently, it is more sensible to consider only differentially expressed set of genes for further comparisons.

We therefore applied FDR ≤ 0.05 (5%) in conjunction with FC (absolute log_2_FC ≥ 0.6) to filter the whole data set. In total, 87 (2%) genes from RNA-seq and 64 (1.5%) from microarray qualified at this cut-off threshold from the 4312 consensus genes (Additional file 1: Table S4). Together, 106 genes satisfied our selection criterion from both the methods (Additional file 1: Table S4). Among them 84 (79.2%) genes were up-regulated, while 22 (20.8%) genes were found to be down-regulated. Further, 45 (~42.45%) genes were common between both the methods, whereas, 42 (39.63%) and 19 (~17.92%) genes were uniquely detected by RNA-seq and microarray respectively (Additional file 1: Table S4; Additional file 2: Figure FS2). We further compared the FC values of the 45 consensus genes both qualitatively and quantitatively. These genes qualitatively agreed 100% by having the same trend of log_2_ transformed FC values by both RNA-seq and microarray (Figure 2D). Likewise the quantitative comparison was performed by estimating the correlation between the magnitude of log_2_FC determined by RNA-seq and microarray for the 45 consensus genes as shown in Figure 2E. The magnitude of FC values between the two methods were found to be well correlated (r_s_ = 0.76, p-value < 0.0001), indicating that the same trend of variation was observed in FC values between the two methods without any dispersion. Thereby, the magnitude of FC values determined by RNA-seq and microarray agreed to a large extent for the 45 consensus genes. In order to further pinpoint the deviation in the FC magnitude quantified by the two methods, we plotted the differences in the FC values determined by RNA-seq with respect to microarray, and the percentage of genes with that difference for the 45 consensus genes (Figure 2F). Majority of the genes (~98%) were found to have a magnitude of FC within the range of ≤ 1.5, while for the remaining 2% of the genes, it was 4.7-times higher in RNA-seq than the microarray based quantification. Based on these comparisons, we concluded that the relative gene expression levels quantified by RNA-seq and microarray were consistent to a large extent for the statistically differentially expressed set of consensus genes.

### Comparison with qRT-PCR

**Figure 3:**
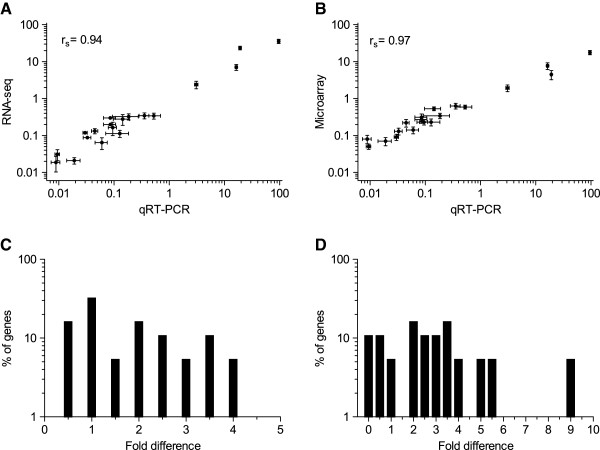
**Comparison of expression levels quantified by RNA-seq and microarray with qRT-PCR. **(**A**) Comparison of expression levels determined by RNA-seq with qRT-PCR. (**B**) Comparison of expression levels determined by microarray with qRT-PCR (**C**) Frequency histogram showing the percentage of genes with the fold difference between RNA-seq and qRT-PCR, with a bin width of 0.5. (**D**) Frequency histogram showing percentage of genes with the fold difference between microarray and qRT-PCR, with a bin width of 0.5.

Traditionally, quantitative Reverse Transcription PCR (qRT-PCR) is used to validate the gene expression levels quantified by high-throughput technologies like RNA-seq and microarray [49]. Therefore, we compared the relative expression levels quantified by RNA-seq and microarray by qRT-PCR for a subset of 43 (40.6%) genes (Additional file 1: Table S5) that were randomly selected from the 106 significantly differentially expressed genes. Among them, 19 genes were found to be common between both the methods, 12 genes were unique to RNA-seq, while remaining 12 genes were found to be unique to microarray (Additional file 1: Table S4). The expression levels were found to be highly reliable for genes that are determined to be significantly differentially expressed by RNA-seq (r_s_ = 0.94; p-value < 0.0001) as well as microarray (r_s_ = 0.97; p-value < 0.0001). For the consensus genes, microarray had a slightly higher correlation with qRT-PCR than RNA-seq (Figures 3A and 3B).

We further plotted the percentage of genes that deviated in the magnitude of FC quantified by RNA-seq and microarray with respect to qRT-PCR (Figures 3C and 3D). For most of the genes, the magnitude of FC quantified by RNA-seq and microarray were relatively higher, when compared to qRT-PCR (fold difference >1). Overall, the magnitude of FC quantified by RNA-seq was in consistence with qRT-PCR based quantification (Figure 3C). For microarray, the magnitude of FC was observed to be consistent with qRT-PCR for a majority of genes, however, we also noticed outlier genes with a 9-times higher FC magnitude (Figure 3D).

**Figure 4:**
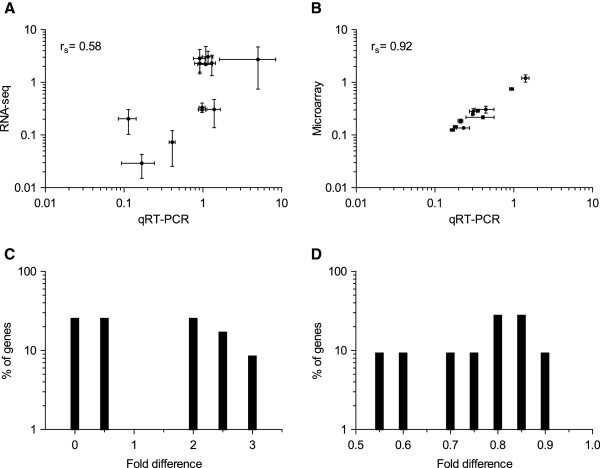
**Comparison of expression levels of genes that are uniquely determined by RNA-seq and microarray with that of qRT-PCR. **Expression levels for the set of selected genes quantified by (**A**) RNA-seq, and (**B**) microarray with that of qRT-PCR. Frequency histogram showing percentage of genes deviating from the magnitude of FC, quantified by RNA-seq (**C**), and microarray (**D**) with respect to qRT-PCR. Bin width of 0.5 and 0.05 are used respectively.

For the subset of 12 genes that were found to be uniquely determined by RNA-seq, the magnitude of FC quantified by RNA-seq correlated moderately with qRT-PCR (r_s_ = 0.58; p-value 0.05) (Figure 4A; Additional file 1: Table S5). The 12 genes found to be uniquely detected by microarray had a correlation of r_s_ = 0.92 (p-value 0.002) with qRT-PCR (Figure 4B; Additional file 1: Table S5). These correlations are slightly lower when compared to the consensus genes (r_s_ ≥ 0.94). This indicated that the expression levels are more reliable for the genes that are determined to be significantly differentially expressed by both RNA-seq and microarray rather than by any one method. Moreover, it also indicated that there is a lot of variation in the magnitude of FC quantified by RNA-seq and qRT-PCR. We further evaluated this variation i.e. deviation from the magnitude of FC, by plotting the frequency histogram for the 12 genes unique to RNA-seq (Figure 4C) and microarray (Figure 4D). For the genes unique to RNA-seq, we observed that none of them had the same magnitude of FC, with 50% genes having 0 to 0.5-time lower and for the remaining 50% of the genes the magnitude of FC was observed to be 2 to 3-time higher, when compared to qRT-PCR (Figure 4C). Because of this inconsistence in the magnitude of FC, the expression levels are moderately correlated. For the genes unique to microarray, we observed a good consistence in the magnitude of FC with qRT-PCR (Figure 4D).

### Comparison in terms of detection of genes encoding T3SS and effectors

Extensive and detailed studies have been carried out since past three decades in cataloguing the target genes of HrpX in the genus Xanthomonas using various genetic and biochemical methods [32,38,39,50-55]. HrpX is known to regulate *hrp* gene cluster that encodes the type III secretion system (T3SS) and effectors [31,56]. T3SS are specialized macromolecular machinery that act as a nano-injector to translocate the effector proteins into the cytoplasm of host plant cells [50]. These translocated effectors manipulate the host cellular processes by altering signal transduction, transcriptional activities like suppression of basal plant defense responses, and protein turnover in host cells for the benefit of the pathogen [50]. The T3SS machineries are evolutionarily conserved across many Gram-negative animal- and plant-pathogenic bacteria [57].

**Figure 5:**
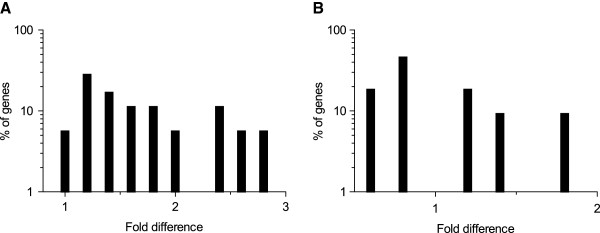
**Comparison of expression levels of genes encoding T3SS and effectors that are commonly detected by RNA-seq and microarray. **(**A**) Frequency histogram showing percent of genes deviating from the magnitude of FC quantified by RNA-seq with respect to microarray for *hrp* gene cluster. Bin width of 0.2 is used. (**B**) Frequency histogram showing percentage of genes deviating from the magnitude of FC quantified by RNA-seq with respect to microarray for the T3SS and effector genes. Bin width of 0.5 is used.

Xcc is comprised of 25 *hrp* genes, including 19 *hrp*-conserved (*hrc*) and 6 *hrp*-associated (*hpa*) genes that encode the T3SS [58]. These genes are clustered in a ~25 kb region spanning from 462712 to 488334 bp of the genome [32]. We applied statistically significant differentially expressed gene list that were derived from RNA-seq and microarray methods to this cluster. We counted for the number of known *hrp* cluster genes, which passed the FC and FDR cut-off thresholds from RNA-seq and microarray methods (Table 1). Among the 25 *hrp* cluster genes, 16 (64%) were detected by both RNA-seq and microarray methods. Six genes were found to be uniquely detected by microarray, whereas, none uniquely detected by RNA-seq (Table 1). Three genes namely, *hrcC*, *hpa2*, and *hpaA* could not pass our statistical cut-off criteria by any of the methods, although they followed the same qualitative expression pattern. We further quantified the deviation in the magnitude of FC for the 16 known *hrp* genes, found in consensus between RNA-seq and microarray (Figure 5A). The magnitude of FC for 5% genes found to be same, while for the remaining 95% genes it was found to be between 1.2 to 1.8-time higher in RNA-seq than in microarray. Even though, microarray overall detected more genes from *hrp* cluster, RNA-seq reported higher magnitude of FC (Table 1).

**Table 1:** **Summary of Type III secretion system (T3SS) *hrp *** **cluster genes detected by RNA-seq and microarray**

**Locus Tag**	**Gene Symbol**	**RNA-seq**			**Microarray**			**Detected by**
		**log**_**2**_**FC**	**p-value**	**FDR**	**log**_**2**_**FC**	**p-value**	**FDR**	
XAC0412	*hrcN*	−2.2355	7.66E-09	1.27E-06	−0.8217	0.00E+00	0.00E+00	†
XAC0409	*hrcJ*	−3.1488	4.20E-15	1.40E-12	−2.1840	0.00E+00	0.00E+00	†
XAC0406	*hrcU*	−2.6729	1.01E-13	2.56E-11	−1.0507	0.00E+00	0.00E+00	†
XAC0405	*hrcV*	−1.5755	3.27E-08	4.88E-06	−0.8427	4.00E-05	2.58E-03	†
XAC0407	*hrpB1*	−3.9638	7.53E-28	3.61E-25	−2.8603	0.00E+00	0.00E+00	†
XAC0408	*hrpB2*	−2.8155	2.48E-09	4.67E-07	−1.9507	0.00E+00	0.00E+00	†
XAC0410	*hrpB4*	−1.9274	7.42E-05	6.05E-03	−1.5237	0.00E+00	0.00E+00	†
XAC0403	*hrcQ*	−2.0615	6.36E-07	7.64E-05	−0.8647	0.00E+00	0.00E+00	†
XAC0402	*hrcR*	−1.7123	4.73E-06	5.53E-04	−0.8677	0.00E+00	0.00E+00	†
XAC0399	*hrpD5*	−1.7287	2.67E-10	5.50E-08	−1.5487	0.00E+00	0.00E+00	†
XAC0398	*hrpD6*	−1.5446	5.85E-07	7.43E-05	−1.6347	0.00E+00	0.00E+00	†
XAC0397	*hrpE*	−2.1535	7.67E-16	2.76E-13	−1.8163	0.00E+00	0.00E+00	†
XAC0394	*hrpF*	−2.0517	7.04E-15	2.17E-12	−1.2113	0.00E+00	0.00E+00	†
XAC0416	*hpa1*	−5.0096	1.49E-51	1.29E-48	−4.2917	0.00E+00	0.00E+00	†
XAC0396	*hpaB*	−1.5429	1.56E-07	2.11E-05	−1.2527	0.00E+00	0.00E+00	†
XAC0393	*hpaF*	−0.9235	1.96E-04	1.34E-02	−0.6083	1.00E-05	8.10E-04	†
XAC0411	*hrpB5*	−1.9690	3.92E-03	1.38E-01	−0.8457	0.00E+00	0.00E+00	ψ
XAC0401	*hrcS*	−1.0156	4.00E-02	4.71E-01	−1.0837	0.00E+00	0.00E+00	ψ
XAC0404	*hpaP*	−1.2673	1.10E-02	2.74E-01	−1.1110	0.00E+00	0.00E+00	ψ
XAC0395	XAC0395	−1.0817	4.73E-02	5.08E-01	−0.8640	0.00E+00	0.00E+00	ψ
XAC0415	*hrcC*	−0.2713	2.49E-01	8.27E-01	−0.4053	4.75E-03	1.78E-01	$
XAC0413	*hrpB7*	−1.2107	1.08E-02	2.74E-01	−0.6590	0.00E+00	0.00E+00	ψ
XAC0417	*hpa2*	−1.4130	8.33E-03	2.31E-01	−0.4610	4.40E-04	2.64E-02	$
XAC0400	*hpaA*	−1.0585	2.76E-03	1.10E-01	−0.7763	9.74E-03	2.05E-01	$
XAC0414	*hrcT*	−1.0474	1.10E-02	2.74E-01	−0.6800	0.00E+00	0.00E+00	ψ

† Consensus between RNA-seq and microarray16 genes (64%).

ξ Only RNA-seq 0 gene (0%).

ψ Only microarray 6 genes (24%).

$ Undetected 3 genes (12%).

The list of known T3SS *hrp* cluster genes along with the information about the log_2_FC, p-value and FDR value from RNA-seq and microarray experiments. "Detected by" column assigns by which method the known *hrp* cluster gene is found to be significantly differentially expressed.

Xcc also encodes 25 putative effector genes regulated by HrpX, which meditate the interaction with the host plant, hence determine the host specificity [55]. Since XAC2785, XAC1210 and XAC1209 were considered as pseudo or inactive genes, they were excluded from our analysis. We tabulated how many of these genes were detected by RNA-seq and microarray methods with their corresponding log_2_FC values along with p-value and FDR from the respective methods (Table 2). In total, 10 (45.5%) genes were detected by both the methods*.* RNA-seq and microarray uniquely detected one and three genes respectively. The remaining 9 genes (36.4%) were neither detected by RNA-seq nor by microarray, since they could not pass both the FC and FDR cut-offs (Table 2). For the 10 consensus genes, we calculated the fold differences in the magnitude of FC quantified by RNA-seq with respect to microarray. None of the genes had the same magnitude of FC between the two methods. Microarray estimated higher magnitude of FC for ~64% genes than RNA-seq, while RNA-seq estimated 1.2 to 1.8-time higher magnitude of FC for the remaining ~36% genes (Figure 5B). In contrast to *hrp* gene cluster, where microarray qualitatively outperformed RNA-seq in its ability to detect more genes, here RNA-seq complemented quantitatively with higher confidence by reporting higher magnitude of FCs. Thereby, for the effector gene data set, RNA-seq and microarray complemented each other both qualitatively as well as quantitatively.

**Table 2:** Summary of Type III effector genes detected by RNA-seq and microarray

**Locus Tag**	**Gene Symbol**	**RNA-seq**			**Microarray**			**Detected by**
		**log**_**2**_**FC**	**p-value**	**FDR**	**log**_**2**_**FC**	**p-value**	**FDR**	
XAC0286	*xopE*	−1.3432	1.21E-07	1.69E-05	−1.1813	0.00E+00	0.00E+00	†
XACb0011	*avrXacE3*	−0.9098	2.20E-04	1.51E-02	−1.6363	0.00E+00	0.00E+00	†
XAC0754	*xopI*	−1.1830	5.40E-04	3.11E-02	−0.7287	0.00E+00	3.00E-05	†
XAC3085	*xopK*	−2.1505	0.00E+00	0.00E+00	−1.7157	0.00E+00	0.00E+00	†
XAC2786	*xopN*	−3.5117	0.00E+00	0.00E+00	−2.9897	0.00E+00	0.00E+00	†
XAC1208	*xopP*	−1.1860	1.00E-05	6.20E-04	−0.7583	0.00E+00	0.00E+00	†
XAC0277	*xopR*	−1.1202	6.00E-05	5.10E-03	−1.2360	0.00E+00	0.00E+00	†
XAC0543	*xopX*	−3.0765	0.00E+00	0.00E+00	−3.4423	0.00E+00	0.00E+00	†
XAC3230	*xopAI*	−1.4353	0.00E+00	0.00E+00	−1.1510	0.00E+00	0.00E+00	†
XAC2922	*hrpW*	−2.0833	0.00E+00	0.00E+00	−2.7723	0.00E+00	0.00E+00	†
XAC4213	*xopAD*	−0.8816	2.60E-04	1.68E-02	−0.3960	2.17E-02	2.72E-01	ξ
XAC4333	*xopQ*	−0.6779	1.95E-02	3.51E-01	−1.1190	0.00E+00	0.00E+00	ψ
XAC0601	*xopV*	−0.5197	5.44E-02	5.33E-01	−0.7367	0.00E+00	2.70E-04	ψ
XAC0076	*avrBs2*	−0.8632	1.79E-03	7.90E-02	−0.566	5.00E-05	3.53E-03	$
XAC3090	*xopL*	−0.3402	2.58E-01	8.37E-01	−0.6040	0.00E+00	0.00E+00	ψ
XAC3224	*xopE*	−0.5716	2.45E-02	3.85E-01	−0.2397	2.68E-02	4.48E-01	$
XAC2009	*xopZ*	−0.6456	9.45E-03	2.56E-01	−0.4160	1.30E-04	9.38E-03	$
XAC3666	*xopAK*	−0.2756	2.89E-01	8.59E-01	−0.2060	3.23E-01	9.82E-01	$
XACa0022	*pthA1*	0.0225	8.00E-01	9.80E-01	0.0790	6.69E-01	9.98E-01	$
XACa0039	*pthA2*	−0.4501	2.09E-01	7.97E-01	0.0413	8.25E-01	9.98E-01	$
XACb0015	*pthA3*	−1.1700	3.52E-03	1.28E-01	0.0723	7.13E-01	9.98E-01	$
XACb0065	*pthA4*	−0.3423	8.51E-01	1.00E+00	0.0315	7.23E-01	9.98E-01	$

† Consensus between RNA-seq and microarray 10 genes (45.5%).

ψ Only microarray 3 genes (13.6%).

ξ Only RNA-seq 1 genes (4.5%).

$ Undetected 8 genes (36.4%).

The list of known T3SS effector genes along with the information about the log_2_FC, p-value and FDR value derived from RNA-seq and microarray experiments. "Detected by" column assigns by which method the following effector genes are found to be significantly differentially expressed.

Overall, considering T3SS and effector genes, in total there are 47 genes, from which, 26 genes (55%) were detected by both RNA-seq and microarray (Tables 1 and 2). RNA-seq uniquely detected 1 gene (2%), whereas, microarray detected 9 genes (19%). Remaining 11 genes (23%) were not detected by either one of the methods by failing to pass the cut-off threshold (Tables 1 and 2). Further, considering only the genes that are detected by at least one method, 72% of the known were detected by both methods, while remaining 28% were detected by either one of the methods.

### Genes uniquely detected by RNA-seq and microarray

Among the 87 statistically significant differentially expressed genes from RNA-seq, 42 (39.63%) genes were found to be uniquely detected by this method (Additional file 2: Figure FS2). Of these 42 genes, 17 were found to be down-regulated, while 25 were up-regulated (Additional file 1: Table S4). Nearly 98% of these genes (41 of 42 unique) could not pass the FC cut-off threshold by microarray. The only exception is the gene *fliO* (XAC1945) that encodes a flagellar protein for flagellum apparatus, which passed the FC cut-off, but failed with FDR threshold. The gene XAC0755 encoding KdpF, a component of an integral membrane potassium-transporting system [59], is down-regulated by a factor of 3 (log_2_ FC of 1.6) according to RNA-seq, but, microarray could not capture this, as the probes for this gene were missing on the chip. This shows the limitation of microarray, where probes for all the genes need to be defined while designing the chip. Furthermore, four genes uniquely found by RNA-seq are involved in signal transduction and gene regulation, i.e. XAC4116 encoding a serine/threonine kinase, XAC1819 encoding a tryptophan-rich sensory protein, and two regulatory genes XAC3026, and XAC3363, whose function in citrus canker disease development remain to be explored. Furthermore, 21 genes (24%) are currently annotated as hypothetical proteins (Additional file 1: Table S6). Among them, four hypothetical proteins XAC0854, XAC4131, XAC1203, and XACb0064 were predicted to be T3SS secreted while 7 hypothetical proteins, XAC3275, XAC3680, XAC1943, XAC0527, XAC0599, XAC0239, and XAC0755 were predicted to be Type 2 Secretion System (T2SS) substrates (Additional file 1: Table S6) by Effective database [60]. Gram-negative bacteria employ T2SS to transport proteins to the extracellular milieu, where the T2SS exo-proteins containing N-terminal signal peptides are used for inner-membrane translocation through either the Sec translocon or the Tat complex [61]. Genes encoding proteins secreted by T3SS and T2SS have been experimentally proved to be regulated by HrpX [33,62,63].

Among the 64 statistically significant differentially expressed genes from microarray, 19 (29.7%) genes were found to be uniquely detected by this method (Additional file 2: Figure FS2). 18 were found to be down-regulated, while one gene was up-regulated (Additional file 1: Table S4). Unlike that of RNA-seq, nearly 63% genes (12 of 19 unique) could pass the FC cut-off threshold, but failed to pass the FDR threshold by RNA-seq. The remaining 37% genes (7 of 19 unique) could not pass both FC and FDR cut-off threshold. Furthermore, six genes were found to be hypothetical. Among them XAC2876, XAC1241, and XAC2370 were predicted as T2SS substrates. XAC1241 predicted as a T2SS substrate, shared 73% identity with a putative secreted protein from *X. campestris* pv*. vesicatoria* strain 85–10. Another T2SS candidate XAC2370 shared 95% identity with a secreted protein from *X. fuscans* subsp. *aurantifolii* str. ICPB 10535. XAC1124 shared 100% identity with MEKHLA domain protein from *X. axonopodis* pv*. punicae* str. LMG 859 [33]. This domain is found in bacteria associated with plants. It further shares similarity with the PAS domain and might be involved in light, oxygen, and redox potential sensation [64].

### Comparison at the level of functional annotations of genes

For comparison based on the biological function for the differentially expressed genes from RNA-seq and microarray, we utilized the ClueGO to integrate the Gene Ontology (GO) [65] terms and KEGG [66] pathway terms and create a functionally organised GO/KEGG network. Functional annotation with biological processes category resulted in 13 (14.94%) genes found from cluster for RNA-seq, while for microarray it was 12 (19.35%).

The ClueGO overview pie chart highlighted that significant proportion of the genes differentially regulated are involved in “protein secretion by the T3SS” by both RNA-seq and microarray (Additional file 3: Figure FS3A & D). Additionally, RNA-seq also identified genes involved in “secretion activity by cell” as well as “single organism catabolic process” (Additional file 3: Figure FS3A). On the other hand, microarray highlighted the genes involved in “protein transmembrane transport”, “polycyclic aromatic hydrocarbon degradation” and “establishment of localization in cell” (Additional file 3: Figure FS3D). Majority of the genes are involved in “bacterial secretion system”, as shown by both RNA-seq and microarray. Also the differentially expressed genes are found to be significantly involved in the “transport of monovalent inorganic cation” (Additional file 3: Figure FS3B) and “protein transport” (Additional file 3: FS3E). Genes have also been found uniquely by microarray as significantly involved in “polycyclic aromatic hydrocarbon degradation” (Additional file 3: Figure FS3E). Genes from RNA-seq have been found to be involved in “riboflavin metabolism” as well as “single organism catabolic process” (Additional file 3: Figure FS3B). Further, visualization of the functionally grouped annotation network for the differentially regulated genes derived from RNA-seq (Additional file 3: Figure FS3C) and microarray (Additional file 3: Figure FS3F) methods highlighted the relationships between the terms. RNA-seq highlighted “protein secretion by the T3SS” along with the “small molecule catabolic process”, while microarray reflected “polycyclic aromatic hydrocarbon degradation” and “establishment of localization in cell”, as the most significant terms of the group. This analysis also showed that RNA-seq and microarray together provide more comprehensive functional information than the individual methods.

### PIP box detection

**Figure 6:**
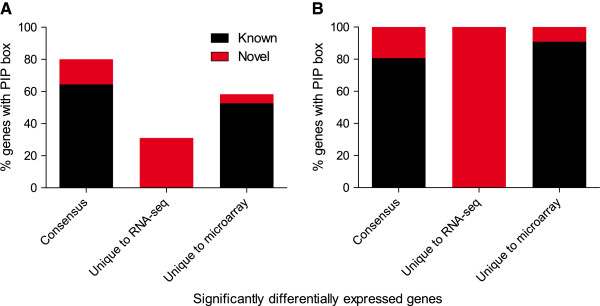
**Statistics of HrpX binding sites in the cis-regulatory regions of significantly differentially expressed genes from RNA-seq and microarray. **The genes belonging to consensus and unique to each method are shown (**A**) Percentage of genes containing PIP box in the cis-regulatory regions of genes that belong to three different groups are shown. The known (black bar) and novel (red bar) are indicated. (**B**) Only genes with PIP box are shown, percentage of which already known (black bar) and novel (red bar) are indicated.

HrpX is known to regulate the target gene expression by specifically binding to PIP box motif present in the cis-regulatory regions. PIP box consists of direct repeats of “TTCGC” with a spacer of 8 to 26-bps in between the repeats, even though ideally 8-bps and 15-bps are considered as the canonical PIP box [37]. We exploited this feature and looked for PIP boxes in the promoter regions of the 106 significantly differentially expressed genes (Additional file 4). All the 106 differentially expressed genes could be assigned to 90 transcriptional units based on MetaCyc database [67] (Additional file 1: Table S8). However for simplicity, genes under the control of the same cis-regulatory regions were counted separately. Among the consensus 45 genes, 36 (80%) were shown to have canonical PIP boxes (Figure 6A, Additional file 1: Table S7). Of the 42 genes that are uniquely determined by RNA-seq, 13 (31%) genes were confirmed to have PIP boxes; whereas, among the 19 genes that are uniquely determined by microarray 11 (57.8%) genes were confirmed to have PIP boxes (Figure 6A, Additional file 1: Table S7). In this study, we identified newly PIP box motif in 7 (19.4%) genes among consensus, 13 (100%) genes unique to RNA-seq and 1 (9%) gene unique to microarray (Figure 6B). Overall, 60 of the 106 (~57%) significantly differentially expressed genes were confirmed to have PIP boxes in their cis-regulatory regions (Additional file 1: Table S7, Additional file 4). The presence of PIP box confirmed that these genes may be directly regulated by HrpX, while the remaining 46 that do not have PIP boxes may be indirectly regulated by HrpX via the other transcription factors. In this regard, we looked for genes with sequence specific DNA binding activity in the 106 differentially expressed genes. Six genes namely *hrpG*, *pcaQ*, *blal*, XAC3026, XAC3445, and XAC3446 were known to have sequence specific DNA binding activity according to GO annotation. Among them, XAC3446, XAC3445, and *blaI* have been newly identified in this study containing PIP box motif (Additional file 1: Table S7, Additional file 4). Thereby these 3 transcription regulators are directly regulated by HrpX, which in turn we assume regulate the 46 genes, which do not contain the PIP box motif and hence indirectly regulated by HrpX. The DNA binding signatures for many of these transcription factors are unknown; hence, obscure the further confirmation of regulation by these transcription factors. Nevertheless, the fact that many of the genes that were uniquely determined by each method showed a clear PIP box in their cis-regulatory regions reiterates that RNA-seq and microarray complement each other.

## Discussion

Currently, RNA-seq is becoming the preferable choice for gene expression profiling in place of microarrays. Although, all the parameters that influence the various aspects of this method are yet to be understood completely, RNA-seq undoubtedly is playing a very important role in deciphering the complexity of the transcriptome by giving a new direction to isoforms, allelic expression, untranslated regions, splice junctions, antisense regulation and intragenic expression [10,16,29,68-74]. Several studies have begun to investigate on the parameters like sequencing depth, precision, GC bias, length bias, lane effects, and processing artifacts [16,29,48,75-77]. On the other hand, microarrays are in usage for more than two decades. Therefore, most of the biases inherent to this method have become more apparent [78]. For instance, biases in the hybridization of the samples labeled with Cyanine5 (Cy5) and Cyanine3 (Cy3) are sufficiently explored, and currently several approaches are practiced to minimize such effects [79-82]. Further, systematic variability like influence of the image scanner settings on the dye intensity measurements have now been robustly handled by applying various normalization techniques [83-86]. Despite these developments, some inherent genes–specific biases like differential hybridization efficiencies of the labeled target transcript to the same probe are still found to be inevitable in microarrays. In RNA-seq as well as microarray, all these known and unknown parameters influence the final outcome. Therefore, in this study, we focused on the assessment of RNA-seq and microarray based on the final outcome .i.e. statistically significant differentially expressed genes.

In comparison with previous RNA-seq studies, with a sequence coverage of 97% we observed for our data set, is in consistence with the reported 89.5% to 95% coverage observed in other bacterial RNA-seq studies [87-89]. In our study, RNA-seq has identified more significantly differentially expressed genes (82%), when compared to microarray (63%) as in previous studies [18,29,30]. The overall correlation (r_s_ 0.76) in the magnitudes of FC for the consensus genes between the two methods was found to be similar or higher than previous studies [18,29,30,72]. Furthermore, our comparison analysis with qRT-PCR suggested that the expression levels were highly reliable for those genes that were determined to be differentially expressed by both RNA-seq and microarray. Hence, confirming the differential expression of genes by multiple methods reduces false positives thereby enhances the biological discovery.

Even though microarray overall outperformed RNA-seq by detecting more known HrpX target genes from the T3SS in *hrp* cluster by satisfying both FC and FDR cut-off threshold, in principle RNA-seq also detected genes *hrpB5, hrcS, hpaP*, XAC0395, *hrpB7*, and *hrcT*, in terms of FC, but failed to pass FDR threshold. This parameter is more directly influenced by error model considered in the statistical method that is used to infer the differential expression rather than RNA-seq itself. For the same read counts, one can get slightly different FDR values depending on the statistical method [90]. But the implementation of all the statistical methods is not feasible for every dataset. From the T3SS in *hrp* cluster, three genes namely, *hrcC*, *hpa2*, and *hpaA* were not found to be detected by both RNA-seq and microarray, mainly because they fail to pass FDR threshold. Interestingly, our previous microarray analysis confirmed that all these three genes are regulated by HrpX, but only at a later stage of the growth phase by satisfying both FC and FDR cut-off thresholds [33]. This consolidates the regulation of some of the genes at later stages of the growth phase. Further, in case of Type III effector genes, 8 genes (36.4%) were not detected by both RNA-seq and microarray within considered cut-off threshold limit. However, among them *xopL*, *avrBs2*, *xopAK* and *xopZ* were found to be regulated by HrpX only at the later stage of the growth phase (OD_600_ time point 0.5), according to our previous microarray analysis [33]. Further, four genes namely, *pthA2*, *pthA1*, *pthA3*, *pthA4* were regulated by another transcription regulator HrpG at early stage of growth phase (OD_600_ = 0.25 and 0.4) as observed in our previous study, while another undetected gene *xopE* was found to be also regulated by HrpG, but only at OD_600_ = 0.25 time point of growth phase [33]. Thereby this study further validated our previous results. Subsequently, both methods detected 100% of the genes known to be regulated by HrpX (at time point OD_600_ = 0.4) without any false positives. Among them, 72% were detected by both the methods while interestingly 28% of the known target genes were detected by either one of the methods. Hence, both the methods together could complement each other.

In addition 55 genes (~51%) were newly identified as differentially expressed by applying both microarray as well as RNA-seq methods, thereby adding up to the already existing repertoire of HrpX regulated genes. Furthermore, 46 (83.6%) genes among them were uniquely identified by either one of the methods. Overall, 21 newly identified genes were found to have PIP box in their promoter regions, wherein 14 (58.3%) genes were uniquely identified by either RNA-seq or microarray. The presence of the PIP box in the promoter regions of the HrpX-regulated genes uniquely identified by RNA-seq and microarray further not only confirmed that these genes are directly regulated by HrpX, but also that these candidates are not false positives. Consequently, 100% of the known HrpX regulated genes could only be detected together by both the methods, since each method missed out on some of the known genes; hence both the methods together enhance the understanding of HrpX regulome by providing a more comprehensive picture.

## Conclusions

This study has significantly advanced our understanding of the regulome of the critical transcriptional factor HrpX and demonstrates that RNA-seq and microarray complement each other in transcriptome profiling. Consequently, our study demonstrates the advantage of applying multiple transcriptome profiling methods to reveal a more comprehensive picture of a transcriptome, rather than relying solely on one method.

## Methods

### Bacterial strains and growth conditions

The wild-type *X. citri* subsp. *citri*[32]*,* and the *hrpX* mutant strains used in this study were described in our previous study [33]. Both the strains were grown at 28°C in nutrient broth (NB), on nutrient agar (NA), or in NYG medium [91]. Antibiotics rifamycin and kanamycin were added to the media at 50 μg/ml final concentrations.

### RNA extraction

Total RNA was extracted from the wild-type and the *hrpX* mutant strains as described in our previous study [33]. Briefly, strains from NA plates were grown in NB medium at 28°C until mid-exponential phase. Cultures were harvested by centrifugation and inoculated in to nutrient-deficient XVM2 medium, after washing the pellet once with the same medium. Cultures were finally harvested for RNA extraction, when the optical density at 600 nm reached the value of 0.4, and mixed immediately with RNAprotect bacterial reagent (Qiagen, Valencia, CA, and U.S.A.). Total RNA was extracted from each replicate separately using RiboPure bacteria kit (Ambion, Austin, TX, USA), according to manufacturer’s instructions. Genomic DNA contamination from the extracted RNA samples was removed using TURBO DNA-free kit (Ambion). Amount and the quality of the RNA samples was initially determined using NanoDrop™ 1000 spectrophotometer (NanoDrop Technologies, Inc., Wilmington, DE). Samples with absorbency at 260/280 and 260/230 nm ratios > 2 were subjected to further processing. Three biological replicates of the wild-type and the *hrpX* mutant samples were used for RNA-seq analysis.

### Microarray data

The microarray data used in this study was generated during our previous study [33]. Three unique 60-mer oligonucleotide probes were designed for each of the 4,427 protein coding genes of *X. citri* subsp. *citri*[33]. 8-by-15-K DNA microarray chips covering the whole genome were implemented under the Agilent platform. These microarrays were processed at the Interdisciplinary Center for Biotechnology Research Microarray Core Facility, University of Florida. The raw data is available at National Center for Biotechnology Information (NCBI) Gene Expression Omnibus (GEO) data repository under the accession number GSE24016 [33].

### mRNA enrichment and RNA-seq

Total RNA samples were enriched for mRNA, by depleting rRNA using MICROBExpress kit from Ambion following the manufacturer’s instructions. Enriched samples were checked for integrity using Agilent 2100 Bioanalyzer (Agilent Technologies, Santa Clara, CA, USA). RNA samples that passed the quality control were sequenced using the Illumina Genome Analyzer IIx (GAIIx) system by following the standard protocol at the Center for Genome Analysis at Yale University. Real-time analysis and base calling were performed using the CASAVA v1.6 pipeline. The raw sequence data has been submitted to the NCBI Sequence Read Archive and assigned with an accession number SRA052842.

### Reads mapping and statistical analysis

The *X. citri* subsp. *citri* whole genome sequence consisting of one chromosome [GenBank: NC_003919.1], and two plasmids [GenBank: NC_003921.3 and NC_003922.1], along with the annotation information were downloaded from NCBI repository (ftp://ftp.ncbi.nih.gov/genomes/Bacteria/). Quality-filtered reads were aligned on to the genome using CLC Genomics Workbench v4.7.2 (CLC bio, Aarhus, Denmark). Reads uniquely aligned to each gene were tabulated from each replicate separately. Differentially expressed genes were estimated using DESeq package [92], available under the open-source Bioconductor suite of programs [93]. DESeq is a powerful tool to estimate the variance in RNA-seq data and test for differential expression [92]. As an input, DESeq accepts a table of read counts for each gene from different biological replicates, and estimates the differentially expressed genes using negative binomial distribution [92]. Statistically significant differentially expressed genes from both microarray and RNA-seq data were obtained by applying a cut-off threshold of FDR ≤ 0.05 (5%) and an absolute log_2_ fold-change ≥ 0.6.

### Bioinformatics analysis

Similarity searches were performed online using position-specific iterative BLAST (PSI-BLAST) at NCBI site against non-redundant protein database [94]. T3SS and T2SS predictions were performed using Effective database [60]. The promoter regions of the significantly differentially expressed genes were retrieved manually using NCBI genome browser to look for the presence of PIP boxes. The differentially expressed genes were assigned to the transcriptional units by referring to the MetaCyc database [67]. Biological interpretation of the differentially expressed genes was carried out using the ClueGO v1.5 [95], a Cytoscape plug-in [96].

### qRT-PCR

All the qRT-PCR assays were performed as detailed elsewhere [33]. Briefly, gene-specific primers were designed for the selected genes using PrimerQuest^SM^ from Integrated DNA technologies (IDT), Coralville, Iowa (Additional file 1: Table S6). qRT-PCR experiments were performed in triplicates, at least three times for each gene using 7500 fast real-time PCR system (Applied Biosystems, Foster City, CA, USA), using a QuantiTect SYBR green RT-PCR kit (Qiagen) with similar results, by following the manufacturer’s instructions. The relative fold change of target gene expression was calculated using 16S rRNA as an endogenous control with the formula 2^–∆∆CT^[97].

### Data availability

The raw RNA-seq data from this study is deposited at the NCBI sequence read archive (http://www.ncbi.nlm.nih.gov/Traces/sra/sra.cgi), under the accession number SRA052842, while the raw microarray data is available at the NCBI Gene Expression Omnibus (http://www.ncbi.nlm.nih.gov/geo) with the accession number GSE24016.

## Competing interests

We declare no competing interests.

## Authors’ contributions

NW conceived the idea and initiated the study. SK performed analysis and interpretation of the data and wrote the manuscript. QY carried out the PCR experiments. YG participated in the study design, sample and data collection, and data interpretation. NW participated in interpretation of data and writing the manuscript and supervised the overall work. All authors read and approved the final manuscript.

## Supplementary Material

Additional file 1 **The following excel format file contains the following 8 additional tables: Table S1: **Summary of RNA-seq reads from wild-type and *hrpX* mutant strains of *X. citri* subsp. *citri*. Table S2: List of genes that are called by both RNA-seq and microarray. Table S3: List of genes that are uniquely called by RNA-seq and microarray. Table S4: List of statistically significant differentially expressed genes by RNA-seq and microarray filtered by cut-off thresholds. Table S5: List of randomly selected genes for the comparison with qRT-PCR from the statistically significant differentially expressed genes from RNA-seq and microarray. Table S6: Gene specific primers used in qRT-PCR experiment. Table S7: Summary of bioinformatics analysis of statistically significant differentially expressed genes to be part of Type III Secretion System (T3SS) and Type II Secretion System (T2SS) along with the occurrence of PIP box. Table S8: List of 90 transcriptional units from *X. citri* subsp. *citri* to which the 106 differentially regulated genes belong. Click here for file

Additional file 2 **Contains the following two additional figures, Figure FS1: **Venn diagram summarizing genes called by both technologies, when comparison is carried out between the total currently annotated open reading frames (ORFs) available transcripts from the transcriptome of *X. citri* subsp. *citri*. Fold-change values are available from RNA-seq (4323) and microarray (4349). Gene’s called by both technologies are indicated by the overlap between the two circles. 4312 are found in consensus, while 11 and 37 are unique to RNA-seq and microarray respectively. Figure FS2: Venn diagram summarizing genes that are significantly differentially expressed determined by RNA-seq and microarray. Gene’s common to both methods are indicated by the overlap between the two circles. Click here for file

Additional file 3 **Figure FS3 - Comparison at the level of the functional annotations of the significantly differentially expressed genes from RNA-seq and microarray. **GO term and KEGG pathway information enrichment analysis is shown for the genes from RNA-seq (left panel) and microarray (right panel). The overview of the analysis is shown in the form of pie chart for gene set from RNA-seq (A), and microarray (D). The histogram shows the number of genes associated with terms for the genes from RNA-seq (B) and microarray (E). Significantly enriched terms are indicated with ’*’. The terms that are functionally related are shown as a network with terms as nodes and relatedness is indicated with thickness of the edges that is based on their kappa score. The most significant term per group are shown for genes from RNA-seq (C) and microarray (F). Click here for file

Additional file 4 **Figure FS4 - Snapshot of the PIP box motif present in the cis-regulatory region of significantly differentially expressed genes is shown in the context of the whole genome of *X. citri* subsp. *citri*. **The absolute position of each PIP box motif occurrence is shown on the whole genome map along with the −10 ‘TATA’ regions and the gene start site. Click here for file
