# Supplementary figures and images for: RNA-seq and microarray complement each other in transcriptome profiling
Source: BMC Genomics. 2012 Nov 15;13:629. doi: 10.1186/1471-2164-13-629 (PMC3534599; doi:10.1186/1471-2164-13-629)

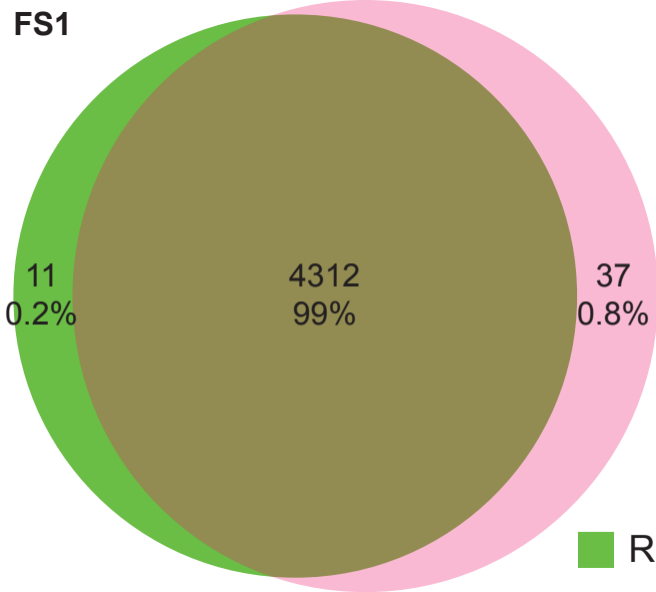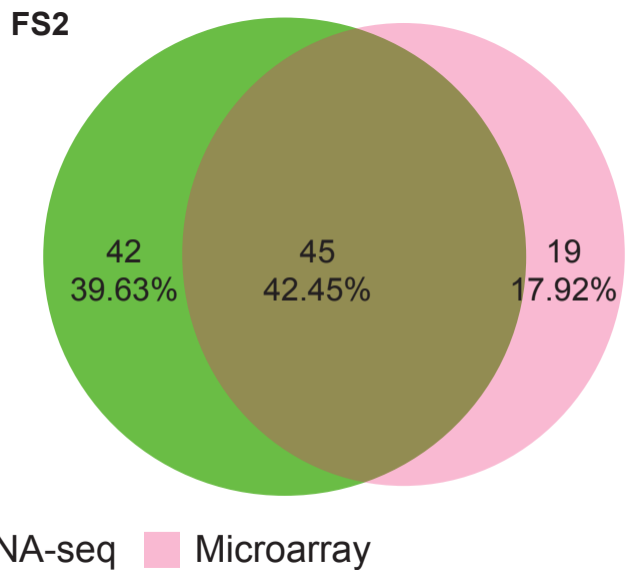

Supplement: Additional file 2 — Contains the following two additional figures, Figure FS1: Venn diagram summarizing genes called by both technologies, when comparison is carried out between the total currently annotated open reading frames (ORFs) available transcripts from the transcriptome of X. citri subsp. citri. Fold-change values are available from RNA-seq (4323) and microarray (4349). Gene’s called by both technologies are indicated by the overlap between the two circles. 4312 are found in consensus, while 11 and 37 are unique to RNA-seq and microarray respectively. Figure FS2: Venn diagram summarizing genes that are significantly differentially expressed determined by RNA-seq and microarray. Gene’s common to both methods are indicated by the overlap between the two circles. [file 1471-2164-13-629-S2.pdf]

A

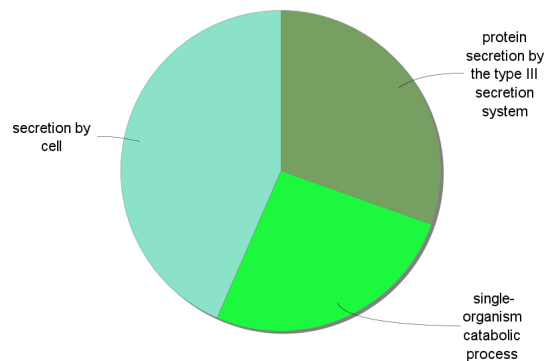

B

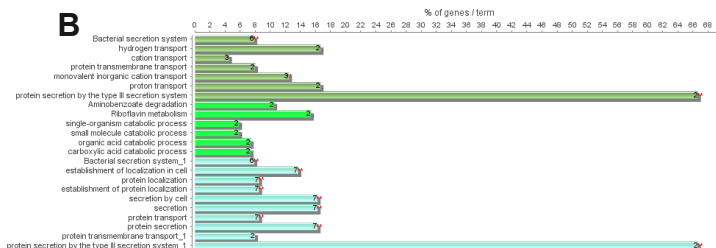

D

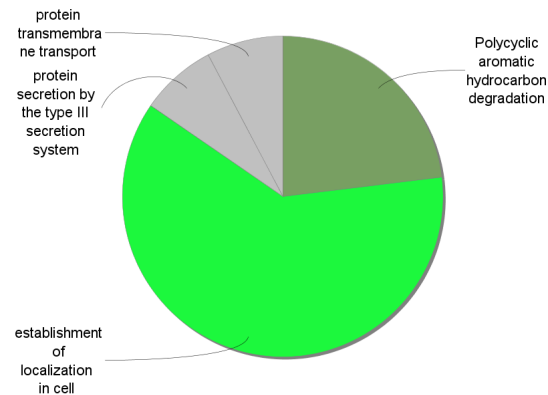

E

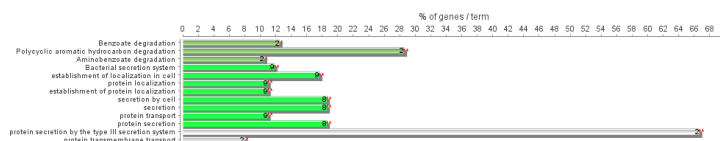

C

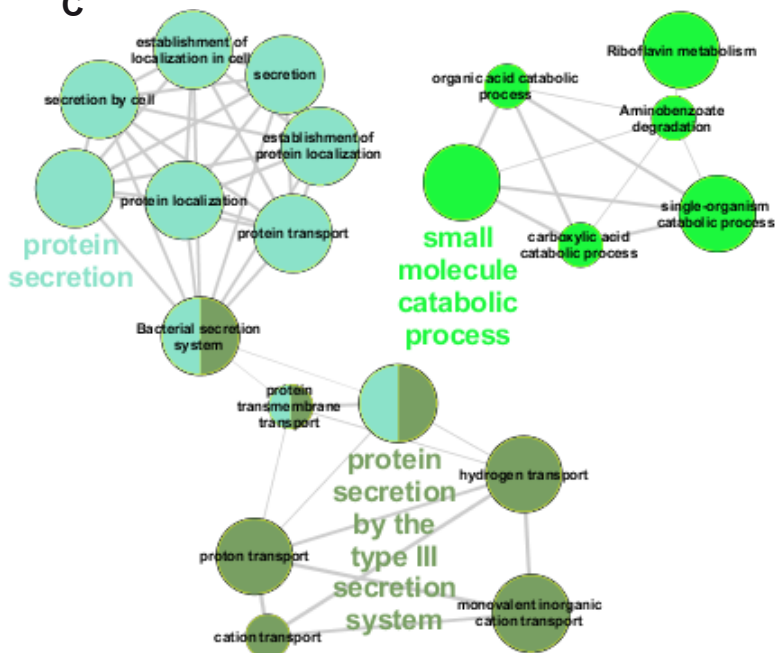

F

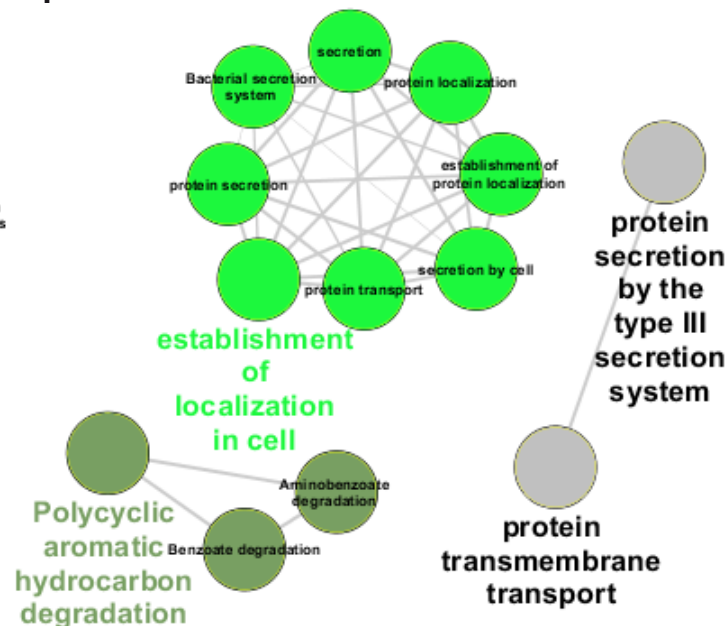

Supplement: Additional file 3 — Figure FS3 - Comparison at the level of the functional annotations of the significantly differentially expressed genes from RNA-seq and microarray. GO term and KEGG pathway information enrichment analysis is shown for the genes from RNA-seq (left panel) and microarray (right panel). The overview of the analysis is shown in the form of pie chart for gene set from RNA-seq (A), and microarray (D). The histogram shows the number of genes associated with terms for the genes from RNA-seq (B) and microarray (E). Significantly enriched terms are indicated with ’*’. The terms that are functionally related are shown as a network with terms as nodes and relatedness is indicated with thickness of the edges that is based on their kappa score. The most significant term per group are shown for genes from RNA-seq (C) and microarray (F). [file 1471-2164-13-629-S3.pdf]

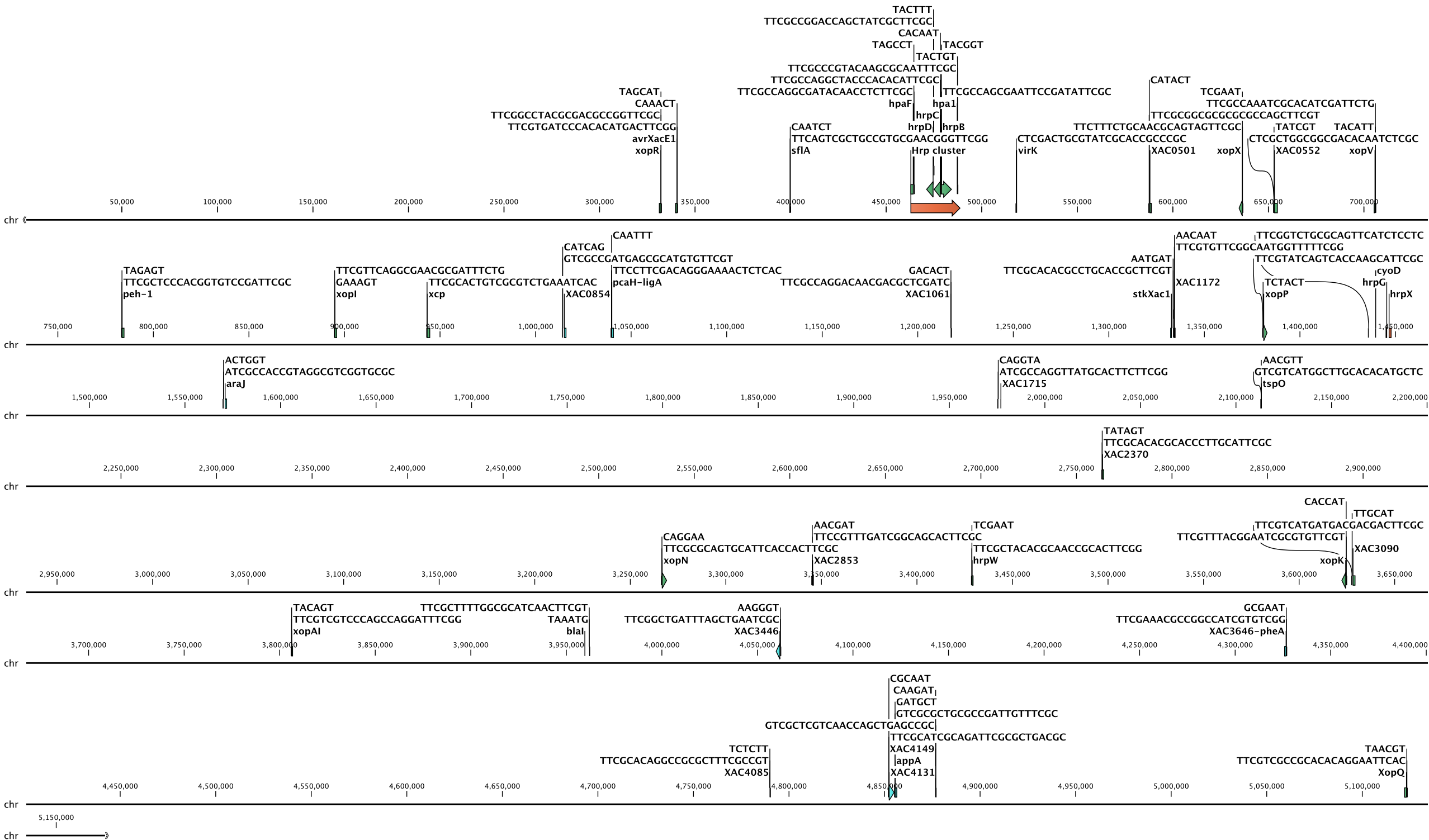

Supplement: Additional file 4 — Figure FS4 - Snapshot of the PIP box motif present in the cis-regulatory region of significantly differentially expressed genes is shown in the context of the whole genome of X. citri subsp. citri. The absolute position of each PIP box motif occurrence is shown on the whole genome map along with the −10 ‘TATA’ regions and the gene start site. [file 1471-2164-13-629-S4.pdf]
